# Cannabidiol versus risperidone for treatment of recent-onset psychosis with comorbid cannabis use: study protocol for a randomized controlled clinical trial

**DOI:** 10.1186/s12888-021-03395-9

**Published:** 2021-08-14

**Authors:** Jesper Østrup Rasmussen, Poul Jennum, Kristian Linnet, Birte Y. Glenthøj, Lone Baandrup

**Affiliations:** 1 Centre for Neuropsychiatric Schizophrenia Research and Centre for Clinical Intervention and Neuropsychiatric Schizophrenia Research, Mental Health Centre Glostrup, Nordstjernevej 41, 2600 Glostrup, Denmark; 2 Danish Centre for Sleep Medicine, Department of Clinical Neurophysiology, University of Copenhagen, Rigshospitalet-Glostrup, Copenhagen, Denmark; 3 Faculty of Health and Medical Sciences, Department of Clinical Medicine, University of Copenhagen, Copenhagen, Denmark; 4 Faculty of Health and Medical Sciences, Section of Forensic Chemistry, Department of Forensic Medicine, University of Copenhagen, Copenhagen, Denmark; 5 Mental Health Centre Copenhagen, Copenhagen, Denmark

**Keywords:** Cannabidiol, Cannabis, THC, Psychosis, Schizophrenia, Dual diagnosis, Randomized clinical trial, Antipsychotic medication

## Abstract

**Background:**

Cannabis use is an important risk factor for development of psychosis and further transition to schizophrenia. The prevalence of patients with psychosis and comorbid cannabis use (dual diagnosis) is rising with no approved specialized pharmacological treatment option. Cannabidiol, a constituent of the *Cannabis sativa* plant, has potential both as an antipsychotic and as a cannabis substituting agent.

The aim of this study is to evaluate the efficacy of cannabidiol versus a first-choice second-generation antipsychotic (risperidone) in patients with early psychosis and comorbid cannabis use.

**Methods:**

The study is a phase II randomized, double-blinded, parallel-group, active-comparator clinical trial. We plan to include 130 patients aged between 18 and 64 years with a recent diagnosis of psychosis, comorbid cannabis use, and currently not treated with antipsychotics. The participants will be randomized to seven weeks of treatment with either cannabidiol 600 mg (300 mg BID) or risperidone 4 mg (2 mg BID). Participants will undergo clinical assessment after 1, 3, 5 and 7 weeks, telephone assessment the weeks in between, and a safety visit two weeks after end of treatment. The primary outcomes are cessation of cannabis use (self-reported) and psychotic symptom severity. The secondary outcomes include frequency and quantity of cannabis use, global illness severity, psychosocial functioning, subjective well-being, cognition, sleep, circadian rhythmicity, and metabolomics.

**Discussion:**

The results of this trial can potentially contribute with a new treatment paradigm for patients suffering from dual diagnosis.

**Trial registration:**

ClinicalTrials.gov, NCT04105231, registered April 23rd, 2021

## Background

Psychotic disorders are characterized by psychotic symptoms including disorganized thinking and speech, delusions and hallucinations [1]. Schizophrenia is the most severe psychotic disorder, characterized by positive (e.g., hallucinations and delusions) and negative symptoms (e.g., lack of initiative and motivation, social withdrawal, emotional blunting) as well as cognitive impairments (e.g., impaired memory and executive functions). Schizophrenia manifests in adolescence or early adulthood in most cases, resulting in marked loss of functioning and reduced quality of life [2]. Antipsychotics are the pharmacological treatment of choice for patients suffering from schizophrenia with clear evidence of symptom reduction and reduced risk of relapse [3]. Dopamine D_2_ receptor blockade is the common mechanism of action of antipsychotics, but antipsychotics also bind to other receptor types with varying affinity [4]. Consequently, antipsychotics are associated with a variety of adverse effects, such as extrapyramidal symptoms, hyperprolactinemia, metabolic disturbances and sedation [4]. Nonadherence toward the medication is a common challenge [3]. A recent review reported that the most important reasons for prematurely stopping therapy included substance abuse (36.1%), a negative attitude toward medication (30.5%) and intolerable adverse effects (27.8%) [3].

Use of cannabis is an important risk factor for development of psychosis and accentuates the severity of psychotic symptoms [1, 5]. The risk of subsequent transition to schizophrenia is associated with the amount and frequency of cannabis used [1, 2]. A recent meta-analysis found that 34% of cannabis-induced psychotic conditions transition to schizophrenia [6]. This was higher than for hallucinogens (26%) and amphetamines (22%). Furthermore, it has been reported that between 19 and 57% of patients with first-episode psychosis use cannabis [2]. The co-existence of psychosis and substance use disorder is commonly referred to as dual diagnosis. In the context of this paper, dual diagnosis denotes a diagnosis of psychosis with comorbid cannabis use. These patients are more likely to be admitted to the hospital, to be hospitalized for longer time periods, to require compulsory admission, and to be prescribed several different antipsychotic medications suggesting an increased risk of treatment failure [7].

People with comorbid substance use disorder are usually excluded from clinical trials which has led to a scarcity of evidence regarding the efficacy of antipsychotics in patients with dual diagnosis. A meta-analysis combining results from trials investigating efficacy of antipsychotics in people with psychosis and substance use disorder found that clozapine, risperidone and olanzapine were slightly superior to other antipsychotics in terms of symptom reduction [8]. Regarding reduction of substance use and craving, clozapine and risperidone were superior to other antipsychotic drugs. The authors reported a similar pattern of adverse effects in this population compared to people without substance use [8].

When reviewing the efficacy of psychosocial treatment for people with severe mental illness and substance abuse, the evidence is sparse and the studies are of low quality [9]. A single study of patients with psychosis and a comorbid use of cannabis found significant effect of motivational interviewing on the amount of cannabis consumed after 3 and 6 months, but this effect was not sustained after 12 months [9].

In the light of the limited treatment data available, best practice today includes integrated psychosis and substance use treatment without advocating for specific treatment practices [10].

### The potential therapeutic benefit of cannabidiol for treatment of dual diagnosis

The *Cannabis sativa* plant contains approximately 100 active compounds (phytocannabinoids). The two major active components are the psychoactive Δ9-tetrahydrocannabinol (THC) and cannabidiol (CBD) [11].

The cannabinoid receptors (CB1 and CB2) are activated by THC and responsible for the psychotomimetic and addictive properties observed after smoking and ingestion of cannabis [12–14]. CBD does not bind to cerebral cannabis receptors and thus has a very different mechanism of action than THC. For the same reason, CBD does not possess euphoric or psychotogenic effects. The cannabinoid receptors are part of the endocannabinoid system, which is also comprised of endogenous cannabinoids and several enzymes which control synthesis and degradation of the endocannabinoids [11]. CBD indirectly affects the endocannabinoid system by impairing the degradation of anandamide (endogenous cannabinoid ligand), which modulates and stabilizes several neurotransmitter systems including the dopaminergic, glutamatergic and GABAergic system [15, 16]. Due to this globally stabilizing effect, CBD theoretically has the potential to reduce craving for cannabis. Likewise, CBD has documented antipsychotic and anxiolytic properties [11, 17, 18], and an anticipated sleep stabilizing effect [19], though the latter has not yet been investigated clinically. Since sleep disturbances play a dominant role regarding the risk of relapse versus remission of psychosis, knowledge about the effect of CBD on sleep disturbances and circadian rhythmicity is a central factor to explore when evaluating the clinical profile of CBD [20]. The pharmacological profile of CBD comprises additionally agonism of 5-HT_1A_ receptors (as many antidepressants), inhibition of re-uptake of adenosine (which plays an important role in the regulation of sleep-wake) as well as antioxidative and anti-inflammatory effects [21].

Cognitive impairment is considered a core symptom of schizophrenia [22]. The potential effect of CBD on cognition has not yet been thoroughly studied. Meta-analyses and reviews have focused on the deleterious effects of THC on cognition, but it has been difficult to study the separate effects of CBD, because THC and CBD are often mixed in different ratios in available cannabis products [23, 24]. Data indicate that CBD counteracts the harmful effects of THC on cognition [23]. THC impairs cognitive functioning in general, but in particular verbal learning, memory and attention [23]. A recent trial investigating CBD as add-on to antipsychotic treatment in patients with schizophrenia found a non-significant increase in cognitive composite score and indication of improvement in motor speed and executive functioning [25].

Taken together, CBD has a multimodal mechanism of action making it potentially superior compared with traditional antipsychotic compounds whose antipsychotic effect is mainly mediated through dopamine D_2_ receptor blockade. The antipsychotic effect of CBD is thought to manifest via inhibition of the degradation of the endocannabinoid anandamide which subsequently antagonizes dopamine D_2_ hyperactivation [11] and thus represents a paradigmatic shift regarding treatment of psychosis.

### Recent evidence

Two published pilot trials have examined the effect of CBD for psychosis. In one study, the effect of CBD was compared with amisulpride for treatment of patients with acute schizophrenia. The results showed that the antipsychotic effect of CBD and amisulpride was comparable, but CBD was associated with significantly fewer adverse effects, such as no increase in prolactin, no weight gain and no extrapyramidal side effects [17]. In another study, CBD was examined as add-on treatment to antipsychotic treatment in patients with schizophrenia, finding a significant reduction in psychotic symptoms together with a favourable side effect profile [25].

Two recent studies investigating the effect of CBD in patients with cannabis use disorder (and no psychotic disorder) found a significant decrease in the amount and frequency of cannabis use compared to placebo [26, 27].

No previous trials have examined the effect of CBD in psychotic patients with comorbid use of cannabis.

### Research objectives and hypotheses

Patients with dual diagnosis can currently be offered few established treatment options. While the development of new antipsychotics in recent years has focused on established mechanisms of action, psychopharmacological alternatives introducing new mechanisms of action and delivering more efficient and better tolerable antipsychotics are urgently required. Based on the studies outlined above, we consider CBD with its putative mechanism of action as one of the most promising candidates to supplement current standards in the therapy of psychosis regarding both efficacy and safety. CBD has unique potential regarding dual diagnosis representing both a promising antipsychotic potential as well as a means to reduce the amount of cannabis use due to its cannabinoid substituting mode of action.

The aim of this study is to evaluate the efficacy of CBD versus a first-choice second-generation antipsychotic (risperidone) in terms of cessation of cannabis use and psychotic symptom reduction in patients with early-stage psychosis and comorbid cannabis use. We hypothesize that CBD will have superior efficacy compared with risperidone for both cessation of cannabis use and symptom reduction.

## Methods

The study is a phase II randomized, double-blinded, parallel-group, active-comparator, clinical trial and planned in accordance with the latest version of The Helsinki declaration [28]. The study is approved by the Danish Ethical Committee of the Capital Region in Denmark (H-19010038), the Danish Data Protection Agency and the Danish Medicines Authority (EudraCT 2018–004893-84).

The trial is registered at ClinicalTrials.gov (NCT04105231), and the conduct of the trial will be in accordance with the protocol. If any necessary modifications of the protocol are made during the trial period, they will be addressed in the publication of the trial results.

### Recruitment

Patients will be recruited from psychiatric hospitals and outpatient clinics under The Mental Health Services in the Capital Region of Denmark, where doctors or nursing staff will inform relevant patients about the project and contact the study team if the patient accepts and might fulfil the eligibility criteria.

Information about the study medication and potential side effects are provided by a research physician or the principal investigator, who will also collect the written informed consent.

The study will be carried out at Mental Health Centre Glostrup that hosts Centre for Neuropsychiatric Schizophrenia Research. This research centre has extensive experience and knowledge in clinical research of psychosis and has an established efficient recruitment set-up.

To ensure steady recruitment to the trial, we will establish a network of committed clinicians from mental health care services across the capital region.

Participants will be compensated for their travel expenses to participate in the present trial. No other economic compensation for study participation will be offered.

When the participants are enrolled in the project, the treatment and monitoring will be carried out in collaboration with the psychiatric hospital or outpatient clinic taking care of the patient before and after participation in the trial. If the participant is not enrolled in an outpatient clinic by the time of inclusion, the project staff will refer the patient to an outpatient clinic upon completion of the trial.

All study participants are covered by the general insurance for patients treated in public hospitals and by the general right to receive compensation for injuries due to pharmaceutical drugs.

### Inclusion criteria

- ICD-10 diagnosis of schizophrenia (DF20.X), paranoid psychosis (DF22.X), acute/intermittent psychotic disorder (DF23.X), schizoaffective psychosis (DF25.X), other/not specified non-organic psychotic disorder (DF28/DF29), or cannabis induced psychotic disorder (DF12.5)
- Within the first 5 years after first-episode psychosis
- PANSS ≥60 and score of ≥4 on ≥2 PANSS-Positive subscale items: Delusions (P1), conceptual disorganization (P2), hallucinatory behaviour (P3), grandiosity (P5), suspiciousness (P6)
- Regular use of cannabis at least on a weekly basis during the past 3 months
- Age 18–64 years
- Female patients of childbearing potential need to utilize a proper method of contraception

### Exclusion criteria

- Treatment resistance as defined by treatment (ever) with clozapine
- Dependence syndrome of alcohol or psychoactive substances (DF1X.2) other than cannabis (DF12.2)
- Psychotic disorder induced by alcohol or psychoactive substances (DF1X.5) other than cannabis (DF12.5)
- Treatment with a long-acting injectable antipsychotic within the past 3 months
- Treatment with an oral antipsychotic within the past 7 days
- Use of self-administered CBD products during the trial
- Patients involuntarily admitted
- Pregnancy or lactation
- Severe physical illness that might influence the ability to comply with the protocol

### Criteria for discontinuation

Relevant information about health status as judged by the investigator after the baseline visit may justify a subsequent exclusion from the study. Relevant information could be information concerning inclusion or exclusion criteria or information that implies an increased risk for the subject in case of participation in the trial.

Reasons for exclusion from the trial:

- Unacceptable adverse effect(s)
- Insufficient effect of treatment on psychotic symptoms with a need of switching to another antipsychotic compound
- Intercurrent physical disease that interferes with the protocol
- Intercurrent pregnancy

Subjects are free to discontinue their participation in the study at any time. Subjects who are not willing to continue the study will be asked about the reason(s) for their discontinuation and about the presence of any adverse events. Dropouts will be seen and assessed by an investigator and the full follow-up test battery will be performed if the participant agrees.

### Experimental intervention and comparison

**Fig. 1:**
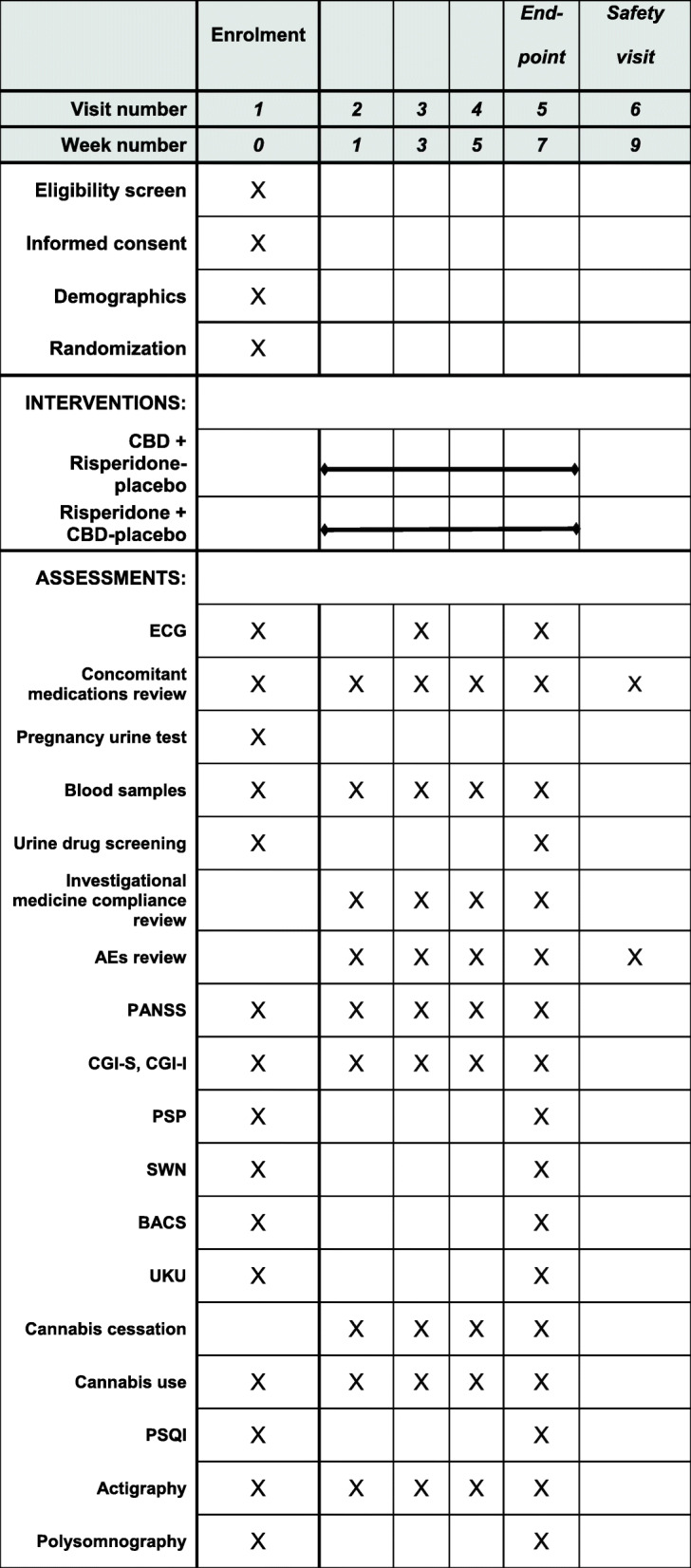
Schedule of enrolment, interventions, visits and assessments. Abbreviations: AE: Adverse event. PANSS: Positive and Negative Syndrome Scale (rating scale - symptom severity). CGI: Clinical Global Impression scale (rating scale – global illness severity), Severity (S) and Improvement (I) scores. PSP: Personal and Social Performance Scale (rating scale – level of psychosocial functioning). SWN: Subjective Well-being Scale under Neuroleptic treatment (rating scale – well-being). BACS: Brief Assessment of Cognition in Schizophrenia (neuropsychiatric test battery). UKU: Udvalget for Kliniske Undersøgelser (rating scale – antipsychotic side effects). PSQI: Pittsburgh Sleep Quality Index (rating scale – subjective sleep quality)

Participants will be randomly assigned in a 1:1 ratio to receive 600 mg of CBD or 4 mg of risperidone, administered in two divided doses morning and evening. Trial duration is seven weeks. After the baseline visit there will be a visit after week 1, 3, 5 and 7 as well as brief evaluations of symptom/disease severity by telephone interview in between, i.e. week 2, 4, and 6. In addition, there will be a safety follow-up visit two weeks after the end of study (week 9). Efficacy will be assessed at the end of the trial, i.e. after seven weeks (Fig. 1).

According to the literature there is no need to titrate the dose of CBD. For risperidone, dose will start at 2 mg and after four days be increased to 2 mg BID. In clinical practice, risperidone is usually titrated within 2–7 days to 4 mg which is a dose that most subjects can tolerate with few adverse effects. To make the groups comparable, the dose of CBD will start at 300 mg once daily and after four days be increased to 300 mg BID. Participants can ingest the total dose once daily if they so prefer. If participants experience intolerable side effects after increasing the dose to two times daily after the first four days, it will be possible to reduce the dose to once daily after discussion and agreement with the investigator.

No other psychotropic medication can be added during the trial except benzodiazepines at a maximum dose equivalent to lorazepam 6 mg daily.

Cannabidiol is delivered as an oral solution with a CBD concentration of 100 mg/ml. Risperidone is administered as encapsulated tablets of 2 mg each. Thus, the CBD solution will be dosed as 3 ml in the morning and 3 ml in the evening, equivalent to CBD 300 mg BID. The risperidone capsules will be dosed as 2 mg in the morning and 2 mg in the evening. Participants are requested to ingest the study medication with a meal to increase the amount of CBD absorbed.

The trial has a double-dummy design, and each group of participants will ingest either CBD solution and risperidone placebo tablets, or risperidone tablets and CBD placebo solution. The CBD solution and the CBD placebo solution will be identical in colour, taste, smell and viscosity, and the same will be true for the encapsulated risperidone tablets and the risperidone placebo capsules.

To enhance adherence to the medication, participants will receive a phone call on day 4 to ensure that the dose is doubled. Adherence will be evaluated by documenting the amount of medication returned at the end of the study and by measuring plasma levels of CBD and risperidone.

### Outcome measures and assessments

#### Primary outcome measures

- *Cannabis cessation* is defined as no use during the past two weeks and is assessed by participant self-report. The self-reported data are the primary outcome, which is in accordance with findings from several studies showing high reliability of self-reported consumption, compared to biometric assessments [29, 30]. The primary outcome will be supported by a sensitivity analysis, considering the concentration of THC in plasma (present or absent) at the end of the study. The concentration of THC in plasma varies in relation to the pattern of cannabis use [29]. With intermittent use, plasma levels mostly decrease within 24 h, but is dependent on the potency of the cannabis used. People with a chronic use can have increased levels for a longer period, in rare cases up to one month [29, 30].
- *Psychotic symptoms* are measured with the Positive and Negative Syndrome Scale (PANSS) [31], using the positive subscale score. PANSS is a rating scale measuring schizophrenia symptom severity based on a semi-structured interview pertaining to the patient’s symptoms during the past week. PANSS is clinician-rated and includes 30 items with a seven-point rating that represents increasing levels of psychopathology. The PANSS includes three subscales: The Positive Scale (7 items), the Negative Scale (7 items), and the General Psychopathology Scale (16 items). The PANSS total and subscale scores are calculated by summing the ratings of all items on the total scale and each subscale (range for total: 30–210, range for Positive and Negative Scales: 7–49, and range for General Psychopathology Scale: 16–112). For this outcome, we use the Positive Scale, and lowest possible score is 7. To avoid an underestimation of the effect, we will, according to Leucht et al. [32], subtract the 7 points from the baseline score when calculating the percentage reduction from baseline.

#### Secondary outcome measures

- *Cannabis use* is assessed by self-reported days of cannabis use per week by use of the time-line follow-back method [33]. To assess the quantity, items from The PSYSCAN Cannabis Questionnaire are used. These items include pictures of different amounts of cannabis to allow for an estimation of the quantity [34].
- *Response* is defined by PANSS total score 25 percentile changes. The results will be presented as 25% percentiles, with proportion of participants with 1–24% PANSS total reduction, 25–49%, 50–74% and 75–100%, as suggested by Leucht et al. [32].
- *Symptomatic remission* is defined according to the Andreasen et al. remission criteria [35]. The criteria define symptomatic remission as a rating of no more than mild in four core positive and four core negative symptoms on the PANSS (P1: Delusions, P2: Conceptual disorganization, P3: Hallucinatory behaviour, N1: Blunted affect, N4: Passive/apathetic social withdrawal, N6: Lack of spontaneity and flow of conversation, G5: Mannerisms and posturing, G9: Unusual thought content) that is sustained for ≥6 months [35]. Because of the duration of this study, the requirement of 6 months duration will not be considered.
- *Global illness severity* is assessed with the Clinical Global Impression Scale (CGI), which is a clinician rated scale that assesses illness severity [36]. The main item ‘severity of illness’ is measured on a 7-point Likert scale (from 1 ‘normal, not at all ill’ to 7 ‘among the most extremely ill patients’). We will use the severity (CGI-S) score at baseline and improvement (CGI-I) scores at the following visits. Response will be defined as much improved or better on the CGI-I.
- *Psychosocial functioning* is assessed using the Personal and Social Performance Scale (PSP) [37]. The PSP is a 100 points single-item clinician-rated scale, divided into four domains: socially useful activities, personal and social relationships, self-care, and disturbing and aggressive behaviours. It has a maximum of 100 points, divided into 10 equal intervals (< 30 = intensive support or supervision needed, 31–70 = disabilities of various degrees, 71–90 = mild difficulties, 91–100 = more than adequate functioning).
- *Neurocognitive functioning* is assessed with the Brief Assessment of Cognition in Schizophrenia (BACS), which is specifically designed to measure treatment-related improvements in patients with schizophrenia [38]. The BACS includes assessments of the following domains: verbal memory, working memory, motor speed, verbal fluency, attention and processing speed and executive function.
- *Well-being* is assessed with the Subjective Well-Being under Neuroleptics Scale, short version (SWN-S) [39]. It is a self-rated 20-item scale consisting of 10 positive and 10 negative items and is rated for the last 7 days. The total score ranges from a minimum of 20 (poor) to a maximum of 120 (excellent).
- *Circadian sleep-wake cycle* is assessed using actigraphy, a wrist-watch-like device, which the participants will wear for the whole study duration. The actigraph measures movement and patterns of movement which are used to estimate circadian rest-activity rhythmicity [40].
- *Subjective sleep quality* is assessed using the Pittsburgh Sleep Quality Index (PSQI) [41]. PSQI is a self-administered rating scale assessing sleep quality and severity of sleep disturbances, rated for the last month at baseline and since last visit subsequently. PSQI consists of 19 items and measures seven components of sleep: sleep quality, sleep latency, sleep duration, habitual sleep efficiency, sleep disturbances, use of sleeping medications, and daytime dysfunction. The component scores each has a range of 0–3 points and they are added to yield one global PSQI score (range of 0–21 points) which distinguishes good sleep (PSQI total score ≤ 5) from poor sleep (PSQI total score > 5).
- *Objective sleep evaluation* is assessed using polysomnography (PSG). PSG is the gold standard for assessing sleep disorders, sleep architecture, and measures of sleep continuity.
- *Metabolomics:*
  - Blood samples are taken at baseline and at each visit to measure plasma levels of cannabinoids (CBD, THC and metabolites), risperidone and metabolomics.
- *Routine safety measures:*
  - HDL, prolactin and liver function tests are measured at baseline and at 7 weeks follow-up.
  - ECG are taken at baseline, after 3 weeks and at 7 weeks follow-up.
  - Data on adverse events are collected at every visit, and by telephone in the weeks between. Patients can reach the researchers at any time in case of spontaneously occurring adverse events.
  - Pregnancy urine test at baseline.

All ratings will be performed by trained clinicians and research nurses.

### Randomization

Central randomization is performed by The Capital Region Pharmacy with computer generated, permuted randomization allocation sequence with block size unknown to the investigator. The Capital Region Pharmacy delivers consecutively numbered and labelled medication packages. When a subject is included, he/she is given the next consecutive corresponding medication package number. The Capital Region Pharmacy delivers sealed envelopes for each randomization number with information of randomization group.

### Blinding

Trial participants as well as trial staff are blinded to the allocated treatment. The blinding will be maintained by using similarly bottled CBD/placebo and similar capsules of risperidone/placebo. The Capital Region Pharmacy will perform the randomization and do the packaging and labelling of trial medication. The Capital Region Pharmacy holds the randomization code which will not be broken until all data are registered, primary analyses finished, and conclusions drawn. The randomization code will only be broken during the trial period in case of emergency if the investigator decides that knowledge about the trial medication will affect the treatment of a serious adverse event (SAE) or in case of a suspected unexpected serious adverse reaction (SUSAR).

### Data collection and managing

Data will be entered in an electronic Case Report Form (eCRF) using the software RedCap which is hosted by the Capital Region and approved as research database by the Danish Data Protection Agency. The trial complies with Danish law on data confidentiality. Data storing and handling is reported to the Danish Data Agency.

RedCap is a web-based and secure database, with real-time entry validation and audit trails. Some clinical data will be entered directly into the eCRF in a RedCap database designed specifically for this study. Other clinical data will initially be registered on paper and then entered manually into the RedCap database. The entered data will be checked by a second person to avoid errors. Study medication will be logged and documented.

Only the investigators will have access to the final dataset. Access to the protocol, data and statistical code can be shared upon request.

Results of this trial, both positive, negative and inconclusive findings, will be published by the investigators in international journals and presented at national and international meetings and conferences.

### Monitoring

The trial will be monitored by the Good clinical Practice (GCP) unit at Copenhagen University Hospital. Trial-related monitoring according to ICH-CGP (International Conference on Harmonisation-Good Clinical Practice) guidelines will be permitted including direct access to the eCRF/source data by the GCP unit. Study monitors from the GCP unit will check that the study procedures are followed correctly and are in accordance with the study protocol.

Due to the short duration of the trial and the minimal risk associated with the intervention, we will not establish a data monitoring committee.

### Statistical analysis

The efficacy of the intervention with respect to the PANSS positive subscale at seven weeks follow-up is analysed using the univariate general linear model with the seven weeks value as the dependent variable (using the last observation carried forward) and the indicator of intervention, age and the baseline value as the independent variables. If the assumptions of the model cannot be fulfilled either directly or after transformation a non-parametric method will be used.

The efficacy of the intervention with respect to the proportion of participants who cease use of cannabis is analysed using a logistic regression model where logit(p) is the dependent variable, p is the probability of cannabis cessation (using the last observation carried forward), and a binary intervention indicator and age are the independent variables.

All analyses will be carried out with the intention-to-treat sample. As secondary analyses the per-protocol sample will be evaluated.

The secondary endpoints will be analysed along the same lines as for the primary efficacy variables.

Subgroup analyses will be performed according to gender.

A sensitivity analysis of the participants with negative plasma-THC at the end of treatment will be carried out.

### Sample size estimation

We consider a relative difference between cannabis cessation proportion in the two intervention groups of 0,50 (i.e. risk ratio for cannabis cessation at follow-up in risperidone group vs. CBD group = 0,5) as a relevant clinical difference. This yields a sample size of 50 patients in each group with a two-tailed 5% significance level and 80% power. Subjects will be randomized in a 1:1 ratio to each intervention group.

We consider that an improvement of 6 points (delta) in the PANSS positive subscale is of minimum clinical relevance (MIREDIF). Leucht et al. defined a clinically meaningful change on the PANSS total scale to be 15 points [42]. Extrapolating this to the positive subscale yields an improvement of 6 points (delta) on the PANSS positive subscale as the MIREDIF. We assume that the PANSS positive score is approximately normally distributed in both treatment groups with a standard deviation of 5 points (sigma) [17]. Thus, with a sample size of 50 subjects, we will be able to detect a difference between groups of 4 points on the PANSS positive subscale, with alfa = 0.05 and a power of 80%.

We expect a drop-out rate between 20 and 30% [43] and thus we reckon that *N* = 130 must be included in the study.

## Discussion

This is the first study to investigate a potential new paradigm in the treatment of patients with psychosis and comorbid cannabis use. Patients with dual diagnosis are particularly difficult to treat and are generally afflicted by worse outcomes than patients with psychosis alone. The increasing potency of available cannabis is of global concern [44, 45], and the results of this trial will become increasingly important.

There is a lack of knowledge on the treatment of patients with dual diagnosis since research so far has focused on patients with psychosis without comorbid substance use disorder.

CBD has been used in trials of animals and humans in up to eight weeks in a dose of 1500 mg/day without complications, toxic effects or major adverse effects, furthermore no physical or psychological dependence was observed [16]. Two published trials have evaluated CBD for treatment of psychosis. In one study, CBD was given in a dose of 800 mg divided in 4 daily doses and was well-tolerated [17]. In another study, a CBD dose of 1000 mg divided in two daily doses was augmented to an existing antipsychotic treatment showing good tolerability [25]. A study investigating the most effective dose of CBD in the treatment of cannabis use disorder found insufficient effect of 200 mg, but significant effect of 400 mg and 800 mg compared to placebo [26]. Thus, we expect a total dose of 600 mg to be sufficient to obtain both acceptable efficacy and tolerability.

The duration of this trial is set to seven weeks. Earlier studies of the efficacy of CBD on psychosis have lasted four to six weeks [17, 25]. Using this duration, it has been possible to show an effect of CBD which was steadily increasing during the entire treatment period but did not find a plateau [17, 25].

The profile of CBD allows for a new and revolutionary paradigm for treating dual diagnosis patients with a potentially substantial impact on the course of illness and the level of functioning. Consequently, CBD has the potential to counteract the transition of acute psychosis to more chronic conditions. The results may have a possible major advantage for current and future patients.

## Trial status

The inclusion begins in May 2021.

Protocol version 4.0 04-12-2020.

## Data Availability

This manuscript does not contain any data. After study completion and after publication of planned primary and secondary analyses the analysed dataset will be available from the corresponding author upon reasonable request.

## Abbreviations

AE: Adverse event
BACS: Brief Assessment of Cognition in Schizophrenia (neuropsychiatric test battery)
BID: Bis in die, twice a day
CB1: Cannabinoid receptor 1
CB2: Cannabinoid receptor 2
CBD: Cannabidiol
CGI: Clinical Global Impression scale (rating scale – global illness severity), Severity (S) and Improvement (I) scores
eCRF: electronic Case Report Form
GCP: Good Clinical Practice
HDL: High-density lipoprotein
ICH-GCP: International Conference on Harmonisation-Good Clinical Practice
MIREDIF: Minimal relevant difference
PANSS: Positive and Negative Syndrome Scale (rating scale - symptom severity)
PSG: Polysomnography
PSP: Personal and Social Performance Scale (rating scale – level of psychosocial functioning)
PSQI: Pittsburgh Sleep Quality Index (rating scale – subjective sleep quality)
SAE: Serious Adverse Event
SUSAR: Suspected Unexpected Serious Adverse Reaction (SUSAR)
SWN: Subjective Well-being Scale under Neuroleptic treatment (rating scale – well-being)
THC: Δ9-tetrahydrocannabinol
UKU: Udvalget for Kliniske Undersøgelser (rating scale – antipsychotic side effects)
